# Real-world evidence on the management and outcomes of glioblastoma in elderly patients: a monocentric retrospective analysis

**DOI:** 10.1186/s12877-026-08457-9

**Published:** 2026-10-13

**Authors:** Jörg Andreas Müller, Ian Schirrwagen, Clara Pitzschel, Daniel Medenwald, Dirk Vordermark

**Affiliations:** 1 Department of Radiation Oncology, University Hospital Halle (Saale), Ernst- Grube-Str. 40, D- 06120 Halle (Saale), Germany; 2 University Clinic for Radiation Therapy, University Hospital Magdeburg A. ö. R, 39120 Magdeburg, Germany; 3 Institute of Computer Science, Martin-Luther-University Halle-Wittenberg, D-06120 Halle (Saale), Germany

**Keywords:** Glioblastoma, Elderly, Survival, Temozolomide, Chemoradiotherapy

## Abstract

**Background:**

Glioblastoma (GBM) carries a poor prognosis, and older patients and those with impaired performance status are often underrepresented in randomized trials. This study evaluated real-world treatment patterns, survival outcomes, and factors associated with outcome in older patients with GBM.

**Methods:**

We retrospectively analyzed 63 patients aged ≥ 65 years with a diagnosis of GBM managed at a single-center radiation oncology department between 2014 and 2023. Overall survival (OS) and progression-free survival (PFS) were calculated from diagnosis. Multivariable Cox models included age, sex, Karnofsky Performance Status (KPS), radiologically confirmed gross total resection (GTR), and temozolomide (TMZ) administration as clinically prespecified covariates. Complete-case analyses were performed. Proportional hazards assumptions were assessed using Schoenfeld residuals, with additional time-varying and exploratory sensitivity analyses addressing diagnostic classification, treatment selection, concomitant TMZ exposure, and treatment timing.

**Results:**

Median OS was 7.0 months (95% CI 5.3–14.4) and median PFS was 5.3 months (95% CI 3.9–7.8). In the multivariable OS model (*n* = 56; 50 deaths), increasing age was associated with higher mortality (HR 1.09, 95% CI 1.02–1.17; *p* = 0.011), whereas female sex was associated with a lower hazard of death (HR 0.45, 95% CI 0.23–0.91; *p* = 0.025). Omission of TMZ was associated with higher mortality (HR 2.26, 95% CI 1.16–4.41; *p* = 0.017), while GTR showed a directionally favorable association that did not reach conventional statistical significance (HR 0.52, 95% CI 0.27–1.01; *p* = 0.054). In the PFS model (*n* = 56; 53 events), increasing age (HR 1.07, 95% CI 1.01–1.14; *p* = 0.027) and omission of TMZ (HR 2.34, 95% CI 1.20–4.55; *p* = 0.013) were associated with higher hazards, whereas female sex (HR 0.45, 95% CI 0.23–0.89; *p* = 0.022) and higher KPS (HR 0.98, 95% CI 0.96–1.00; *p* = 0.039) were associated with lower hazards. Resection status violated the proportional hazards assumption for PFS; time-varying analysis indicated an early favorable association of GTR that attenuated over time. Sensitivity analyses based on concomitant TMZ exposure and survival measured from radiotherapy initiation yielded similar associations.

**Conclusions:**

Survival remained limited in this real-world cohort of older patients with GBM. Age, sex, performance status, resection status, and TMZ administration showed varying associations with outcome, with TMZ showing the most consistent association across analyses. Given the small retrospective cohort and substantial treatment selection, these findings should be considered hypothesis-generating rather than causal.

**Supplementary Information:**

The online version contains supplementary material available at https://doi.org/10.1186/s12877-026-08457-9.

## Introduction

Malignant primary brain tumors, though relatively rare with an annual incidence of approximately 7 per 100,000 individuals, remain among the most devastating cancers, causing more than 15,000 deaths per year in the United States [1]. Glioblastoma (GBM) (WHO grade IV) represents nearly half of all malignant brain tumors and carries a particularly poor prognosis, with a five-year survival rate of only about 6–7% [1].

The classification of adult diffuse gliomas has evolved substantially with the increasing integration of molecular diagnostics. The 2021 WHO Classification of Central Nervous System Tumors introduced an integrated histomolecular framework in which glioblastoma is defined as an IDH-wildtype adult-type diffuse astrocytic glioma fulfilling established histological and/or molecular diagnostic criteria [2, 3]. Molecular alterations therefore play an essential role in distinguishing glioblastoma from other adult-type diffuse gliomas and in defining biologically distinct disease entities [4, 5]. This evolution in diagnostic criteria is particularly relevant to retrospective cohorts spanning different classification eras.

Molecular biomarkers also have important prognostic and predictive implications. In particular, methylation of the MGMT promoter is associated with increased sensitivity to alkylating chemotherapy and has been established as an important predictive biomarker for benefit from temozolomide (TMZ) [6].

The current standard of care consists of maximal safe surgical resection followed by combined radiotherapy (RT) and TMZ, which significantly improves survival compared with RT alone [1, 7]. Additional treatment modalities have been investigated to further improve outcomes. Tumor Treating Fields (TTFields), a non-invasive antimitotic therapy, demonstrated a significant prolongation of both progression-free and overall survival when added to maintenance TMZ in patients with newly diagnosed GBM, with a manageable toxicity profile primarily consisting of mild skin reactions [8]. The vascular endothelial growth factor (VEGF) inhibitor bevacizumab, although widely used in recurrent disease, has not been shown to improve overall survival (OS) on a population level, despite potential benefits such as reduced corticosteroid dependence [9]. Retrospective and registry-based studies confirm the benefit of the Stupp protocol, with OS and PFS compared with pre-2004 cohorts, and have highlighted the potential role of second-line chemotherapy and radiosurgery at recurrence [10].

In elderly patients, therapeutic strategies require further adaptation due to limited tolerance to intensive treatment. Randomized trials have demonstrated that RT provides a modest survival benefit compared with supportive care alone, without impairing quality of life [11]. Moreover, hypofractionated RT regimens are non-inferior to conventional RT and offer advantages such as reduced treatment duration and lower corticosteroid requirements [12]. The addition of TMZ to short-course RT significantly prolongs survival, particularly in patients with MGMT promoter methylation [13]. Similarly, randomized evidence supports both TMZ monotherapy and hypofractionated RT as reasonable treatment options in patients over 70 years, with MGMT methylation status serving as a predictive biomarker for treatment benefit [14].

Several retrospective and population-based studies have further characterized treatment selection and outcomes in older patients with GBM, consistently highlighting the importance of performance status, surgical management, and combined-modality treatment [15–17]. However, substantial heterogeneity remains in treatment patterns and outcomes, particularly among very old patients and those treated outside clinical trials [18, 19]. The present study therefore aimed to provide additional contemporary real-world evidence from a single-center radiation oncology cohort of patients aged ≥ 65 years and to evaluate associations between routinely available clinical and treatment-related factors and OS and PFS.

## Methods

### Data and material

Patients were identified retrospectively from the digital archives of the Department of Radiation Oncology at University Hospital Halle (Saale). Accordingly, the source population comprised patients aged ≥ 65 years with a documented diagnosis of glioblastoma who were referred to and managed by the radiation oncology department between 2014 and 2023 and does not represent an institution-wide registry of all patients diagnosed with glioblastoma. Patients who were not referred for radiation oncology evaluation or who received supportive care alone may therefore not have been captured. Clinical data were extracted from the hospital information system ORBIS (version 03.20.02.01), while imaging and radiotherapy data were obtained from Centricity PACS (GE Healthcare) and Elekta Mosaiq (version 2.84).

Because the study period spanned 2014–2023, diagnostic classification reflected the neuropathological and molecular criteria applicable at the time of diagnosis. No centralized pathological re-review, additional molecular testing, or systematic retrospective reclassification according to the 2021 WHO Classification of Central Nervous System Tumors was performed. Of 71 patient records identified, three patients with documented IDH-mutant tumors and one patient treated with stereotactic radiotherapy were initially excluded. Four additional records were excluded because of irreconcilable chronological inconsistencies in the available retrospective treatment or follow-up data that could no longer be verified from source documentation, resulting in a final primary cohort of 63 patients. Patients with unknown IDH status were retained in the primary real-world cohort. The potential influence of incomplete molecular classification was addressed in an additional sensitivity analysis restricted to patients with confirmed IDH-wildtype disease.

The study was approved by the Ethics Committee of the Medical Faculty of Martin Luther University Halle-Wittenberg (approval number 2024-002).

Clinical variables were categorized for statistical analysis. Age at diagnosis was recorded as a continuous variable and summarized using the median and range. Older age was defined as ≥ 65 years at diagnosis. Sex was classified as male or female. Performance status was assessed using the Karnofsky Performance Status (KPS) and, where available, the ECOG Performance Status. Molecular variables included MGMT promoter methylation status, IDH mutation status, TERT mutation status, and EGFR amplification/mutation.

Surgical management was categorized as gross total resection (GTR), near-total resection (NTR), subtotal resection (STR), biopsy, or no surgery according to the information available in the institutional records. Radiologically confirmed GTR was defined as the absence of detectable residual tumor on postoperative imaging. For survival analyses, all other known surgical categories were combined as non-GTR and served as the reference category, while patients with insufficient information to determine resection status were treated as missing. Three patients were classified as having undergone no surgery in the available radiation oncology dataset. As the original source documentation was no longer available for re-review, it could not be determined whether diagnostic tissue sampling had been performed externally or whether this classification reflected incomplete historical documentation; these cases were therefore retained as a separate descriptive category without retrospective reclassification.

Radiotherapy parameters included fractionation scheme (normofractionated, hypofractionated, or ultrahypofractionated), total prescribed dose, dose per fraction, overall treatment time, and treatment completion. Radiotherapy was considered complete if the full prescribed course was delivered. Specific dose-fractionation schedules were recorded and are reported descriptively.

TMZ exposure was recorded as a binary variable indicating whether TMZ was administered during the documented treatment course and was further classified as concomitant and/or sequential administration. Additional variables included the administered dose and the number of completed treatment cycles. Exact dates of TMZ initiation were not consistently available in the retrospective dataset and therefore did not permit formal modeling of TMZ as a time-dependent exposure.

Dexamethasone use was documented at treatment initiation and at the end of treatment, including the administered dose. Tumor location was recorded according to the anatomical region or regions involved and summarized using absolute and relative frequencies. Tumor volumes were assessed as gross tumor volume (GTV and GTV recurrence) and clinical target volume (CTV and CTV recurrence) and summarized descriptively.

Progression status and the date of progression were based on the contemporaneous clinical assessment documented in multidisciplinary tumor board records and/or follow-up MRI reports. No centralized imaging review or systematic retrospective reassessment according to standardized RANO criteria was performed. Consequently, treatment-related imaging changes, including possible pseudoprogression, could not be systematically reclassified retrospectively. Where sufficient imaging information was available, recurrence was categorized as in-field or out-of-field; otherwise, the recurrence pattern was considered indeterminate.

### Statistical analyses

The primary endpoint was overall survival (OS), and progression-free survival (PFS) was the secondary endpoint. Both endpoints were calculated from the date of diagnosis. OS was defined as the time from diagnosis to death from any cause, with patients alive at last follow-up censored at that date. PFS was defined as the time from diagnosis to the first occurrence of radiologically or clinically documented tumor progression or death from any cause, whichever occurred first; patients without either event were censored at their last known follow-up.

Kaplan–Meier methods were used to estimate survival distributions and median survival with corresponding 95% confidence intervals. For subgroup Kaplan–Meier analyses, unadjusted hazard ratios (HRs) with 95% confidence intervals were derived from corresponding univariable Cox models.

Associations with OS and PFS were assessed using Cox proportional hazards regression. Age, sex, Karnofsky Performance Status (KPS), TMZ administration, and resection status were included in the multivariable models as clinically prespecified covariates irrespective of their statistical significance in univariable analyses. Age and KPS were modeled as continuous variables. Results are reported as HRs with 95% confidence intervals and two-sided p-values.

Missing covariate data were handled using complete-case analysis without imputation. The primary multivariable models included 56 patients, with 50 deaths for the OS analysis and 53 progression or death events for the PFS analysis. One implausible GTV value (9342 cm³) identified during data quality review was treated as missing because the correct value could not be reliably reconstructed.

The proportional hazards assumption was evaluated using scaled Schoenfeld residuals and formal tests for individual covariates and the overall model. Because resection status violated the proportional hazards assumption in the PFS model, an additional Cox model incorporating a time-varying coefficient for GTR was fitted.

Additional exploratory sensitivity analyses were performed to address diagnostic classification, treatment selection, and potential bias related to treatment timing. These included restriction to patients with confirmed IDH-wildtype disease, additional adjustment for radiotherapy regimen, and, among patients with known MGMT promoter status, additional adjustment for MGMT methylation. To address the definition and timing of TMZ exposure, additional models classified exposure according to concomitant TMZ administration. Furthermore, OS and PFS were re-anchored at the date of radiotherapy initiation as an additional sensitivity analysis addressing potential treatment-timing bias. Because resection status continued to show non-proportional hazards in PFS sensitivity models, corresponding models incorporated a time-varying coefficient for GTR. Results of the concomitant-TMZ and radiotherapy-start analyses are reported in Supplementary Table S5. Given the sample size and observational design, all sensitivity analyses were considered exploratory.

Given the observational study design, associations between treatment variables and outcomes were interpreted as non-causal. All statistical analyses were conducted using RStudio, version 2024.04.2 + 764. A two-sided p-value < 0.05 was considered statistically significant.

### Use of AI-assisted technologies

During the preparation of this manuscript, the authors used ChatGPT (OpenAI) to improve writing style, grammar, and language clarity. All generated content was reviewed and edited by the authors, who take full responsibility for the final manuscript.

## Results

### Patient characteristics

A total of 63 patients aged ≥ 65 years with a diagnosis of glioblastoma were included in the primary analysis (Table 1). The median age at diagnosis was 74.0 years (range 65–88 years). Thirty-six patients (57.1%) were male and 27 (42.9%) were female. The median Karnofsky Performance Status (KPS) was 60% (range 30–100%), with data available for 58 patients.

**Table 1:** Baseline clinical, molecular, and treatment characteristics of patients with glioblastoma

Characteristic	*N* = 63
Age, years, median (range)	74.0 (65–88)
Sex	
Male	36 (57.1%)
Female	27 (42.9%)
Karnofsky Performance Status, %, median (range)	60 (30–100); *n* = 58
MGMT promoter status	
Unmethylated	22 (34.9%)
Methylated	34 (54%)
Unknown	7 (11.1%)
IDH status	
Wild-type	52 (82.5%)
Unknown	11 (17.5%)
Surgical management	
Radiologically confirmed GTR	17 (27%)
Near-total resection (NTR)	22 (34.9%)
Subtotal resection (STR)	2 (3.2%)
Biopsy	16 (25.4%)
No surgery	3 (4.8%)
Unknown	3 (4.8%)
Radiotherapy regimen	
Normofractionated	15 (23.8%)
Hypofractionated	41 (65.1%)
Ultrahypofractionated	7 (11.1%)
Total radiotherapy dose, Gy, median (range)	40.0 (5–60); *n* = 62
Radiotherapy completion	
Completed	54 (85.7%)
Not completed	9 (14.3%)
Temozolomide treatment pattern	
No TMZ	22 (34.9%)
Concomitant only	17 (27%)
Concomitant + sequential/adjuvant	19 (30.2%)
Sequential/adjuvant only	4 (6.3%)
Concomitant; sequential status unknown	1 (1.6%)
GTV, cm³, median (IQR)	40.1 (17.9–62.2); *n* = 55
CTV, cm³, median (IQR)	142 (95.5–175); *n* = 41
Recurrence pattern	
In-field	3 (4.8%)
Out-of-field	14 (22.2%)
Unknown / indeterminate	46 (73.0%)
Radiologic residual tumor	
No	17 (27%)
Yes	34 (54%)
Unknown	12 (19%)
Tumor Treating Fields (TTFields)	
No	52 (82.5%)
Yes	5 (7.9%)
Unknown	6 (9.5%)

Data are presented as median (range) for age, KPS, and radiotherapy dose; n (%) for categorical variables; and median (interquartile range) for GTV and CTV. Available-case sample sizes are shown for KPS, radiotherapy dose, GTV, and CTV. Percentages use *N* = 63 unless otherwise indicated. For Cox regression analyses, radiologically confirmed GTR was compared with all other known surgical categories (non-GTR); patients with unknown resection status were excluded by complete-case analysis. GTR, gross total resection; NTR, near-total resection; STR, subtotal resection; GTV, gross tumor volume; CTV, clinical target volume; TTFields, Tumor Treating Fields

MGMT promoter status was methylated in 34 patients (54.0%), unmethylated in 22 (34.9%), and unknown in 7 (11.1%). Fifty-two patients (82.5%) had documented IDH-wildtype disease and 11 (17.5%) had unknown IDH status.

Regarding surgical management, 17 patients (27.0%) had radiologically confirmed GTR, 22 (34.9%) were classified as NTR, 2 (3.2%) as STR, 16 (25.4%) underwent biopsy, and 3 (4.8%) were classified as having no surgery. Surgical status was unknown in 3 patients (4.8%).

Radiotherapy was delivered as hypofractionated treatment in 41 patients (65.1%), normofractionated treatment in 15 (23.8%), and ultrahypofractionated treatment in 7 (11.1%). The most frequently prescribed dose-fractionation schedules were 40.05 Gy in 15 fractions (*n* = 33), 60 Gy in 30 fractions (*n* = 10), 25 Gy in 5 fractions (*n* = 5), and 45 Gy in 15 fractions (*n* = 5). The exact planned dose-fractionation schedule could be derived for 61 of 63 patients. The median administered radiotherapy dose was 40.0 Gy (range 5–60 Gy; *n* = 62). Overall, 54 of 63 patients (85.7%) completed the prescribed radiotherapy course.

TMZ was not administered in 22 patients (34.9%). Seventeen patients (27.0%) received concomitant TMZ only, 19 (30.2%) received concomitant followed by sequential/adjuvant TMZ, and 4 (6.3%) received sequential/adjuvant TMZ without documented concomitant treatment. In one patient (1.6%), concomitant TMZ was documented but subsequent sequential treatment status was unavailable.

Volumetric data were available for 55 patients for GTV and 41 patients for CTV. Median GTV was 40.1 cm³ (IQR 17.9–62.2), and median CTV was 142.0 cm³ (IQR 95.5–175.0).

The recurrence pattern was classified as in-field in 3 patients (4.8%) and out-of-field in 14 (22.2%); it was unknown or indeterminate in 46 patients (73.0%). Radiologic residual tumor was documented in 34 patients (54.0%), absent in 17 (27.0%), and unknown in 12 (19.0%). Tumor Treating Fields were initiated in 5 patients (7.9%), whereas 52 (82.5%) did not receive TTFields and status was unknown in 6 (9.5%). Baseline differences according to TMZ administration are summarized in Supplementary Table S1.

Women had a lower median KPS than men (60% vs. 70%; *p* = 0.043), whereas age did not differ significantly by sex (median 74.0 vs. 73.5 years; *p* = 0.102). Among patients with known MGMT promoter status, methylation was present in 18 of 27 women (67%) and 16 of 29 men (55%), with no statistically significant association between sex and MGMT methylation status (Fisher’s exact test, *p* = 0.423). All seven patients with unknown MGMT status were male. TMZ was omitted in 44% of women and 28% of men (*p* = 0.192), while no statistically significant differences were observed for radiotherapy regimen (*p* = 0.748) or resection status (*p* = 0.145). Selected clinical characteristics according to sex are summarized in Supplementary Table S2.

### Survival analyses

**Fig. 1:**
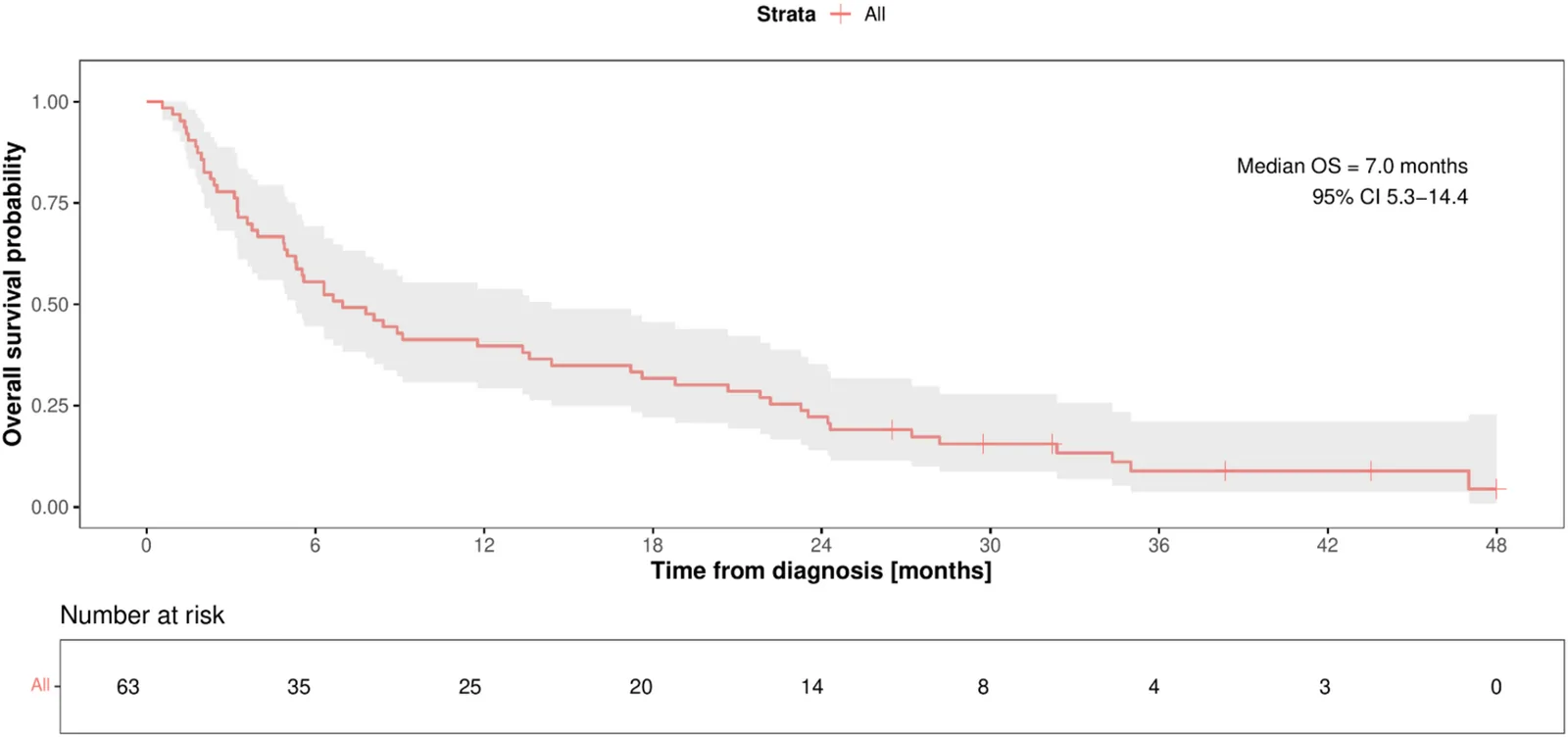
Kaplan-Meier estimate of overall survival in the entire cohort**.** Overall survival (OS) was measured from the date of diagnosis to death from any cause, with surviving patients censored at last known follow-up. The median OS was 7.0 months (95% CI 5.3–13.4). The shaded area represents the 95% confidence interval, censoring is indicated by crosses, and the number at risk is shown below the plot

**Fig. 2:**
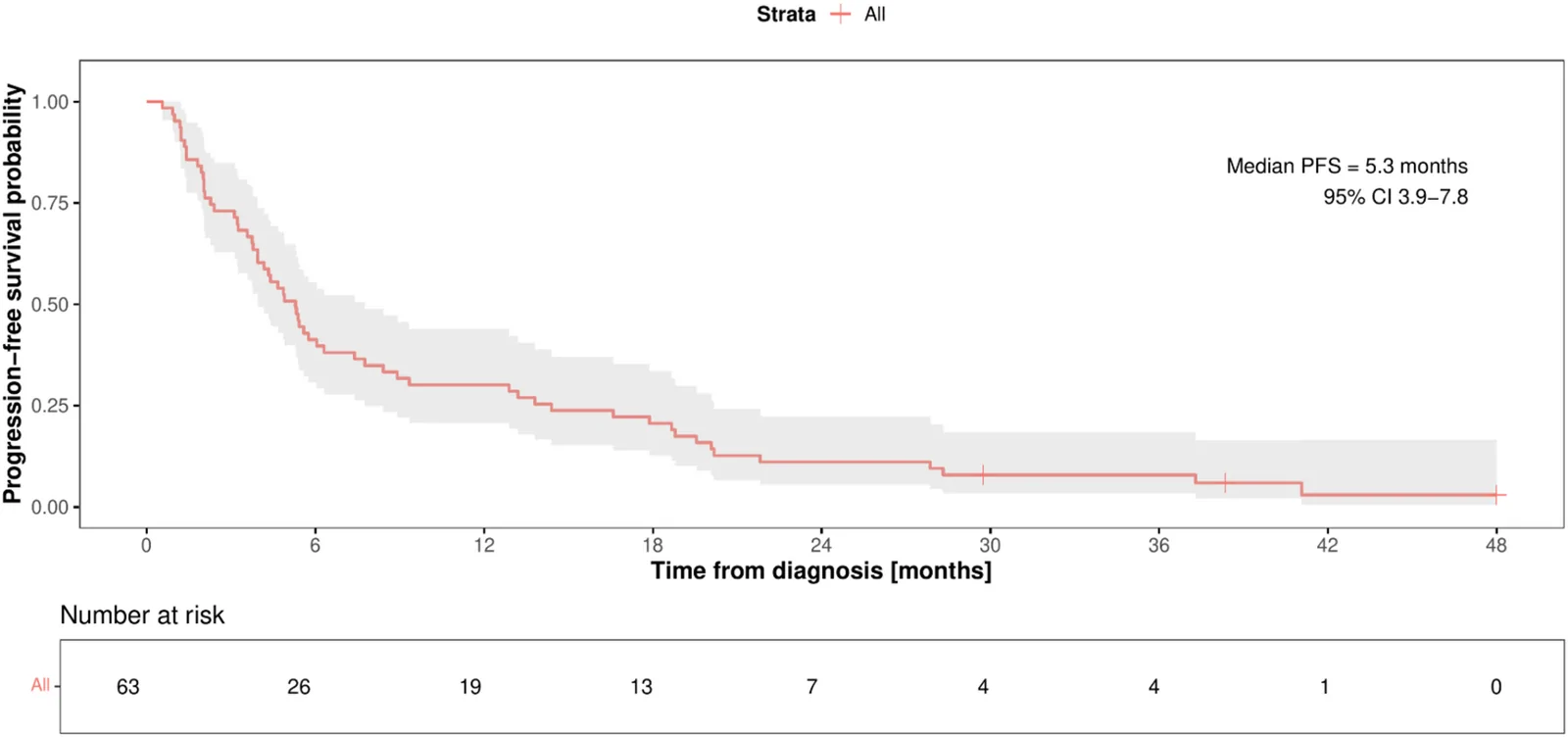
Kaplan-Meier estimate of progression-free survival in the entire cohort. Progression-free survival (PFS) was measured from the date of diagnosis to the first occurrence of documented disease progression or death from any cause; patients without either event were censored at last known follow-up. The median PFS was 4.9 months (95% CI 3.9–6.4). The shaded area represents the 95% confidence interval, censoring is indicated by crosses, and the number at risk is shown below the plot

During follow-up, 57 deaths and 60 PFS events were observed among 63 patients. Median OS was 7.0 months (95% CI 5.3–14.4), and median PFS was 5.3 months (95% CI 3.9–7.8) (Figs. 1 and 2). Of the 60 PFS events, 32 represented documented disease progression and 28 represented death without previously documented progression. Among the patients with documented progression, the anatomical recurrence pattern relative to the radiotherapy field could be classified in 17 patients; the remaining cases lacked sufficient information for reliable in-field versus out-of-field classification.

Univariable and multivariable Cox regression results for OS are summarized in Table 2. The prespecified multivariable model included 56 patients and 50 deaths. Increasing age was associated with higher mortality (HR 1.09 per year, 95% CI 1.02–1.17; *p* = 0.011). Female sex was associated with a lower hazard of death (HR 0.45, 95% CI 0.23–0.91; *p* = 0.025). Omission of TMZ was associated with a higher mortality hazard (HR 2.26, 95% CI 1.16–4.41; *p* = 0.017). Higher KPS was not significantly associated with OS (HR 0.98 per point, 95% CI 0.96–1.00; *p* = 0.114). GTR showed a directionally favorable association that did not reach conventional statistical significance (HR 0.52, 95% CI 0.27–1.01; *p* = 0.054).

**Table 2:** Univariable and multivariable Cox regression analyses for overall survival

Characteristic	Univariable			Multivariable		
	HR	95% CI	*p*-value	HR	95% CI	*p*-value
Age	1.09	1.03–1.15	0.002	1.09	1.02–1.17	0.011
Female sex	0.72	0.41–1.27	0.252	0.45	0.23–0.91	0.025
KPS	0.98	0.96–1.00	0.016	0.98	0.96–1.00	0.114
No TMZ	2.80	1.53–5.11	< 0.001	2.26	1.16–4.41	0.017
GTR	0.61	0.33–1.14	0.123	0.52	0.27–1.01	0.054

HR, hazard ratio; CI, confidence interval; KPS, Karnofsky Performance Status; TMZ, temozolomide; GTR, gross total resection. Univariable and multivariable analyses were performed in the same complete-case cohort (*N* = 56; 50 deaths). The multivariable model included age, sex, KPS, TMZ administration, and resection status irrespective of univariable statistical significance. HRs for age and KPS are reported per 1-year and 1-point increase, respectively. Reference categories were male sex, TMZ administration, and non-GTR

**Fig. 3:**
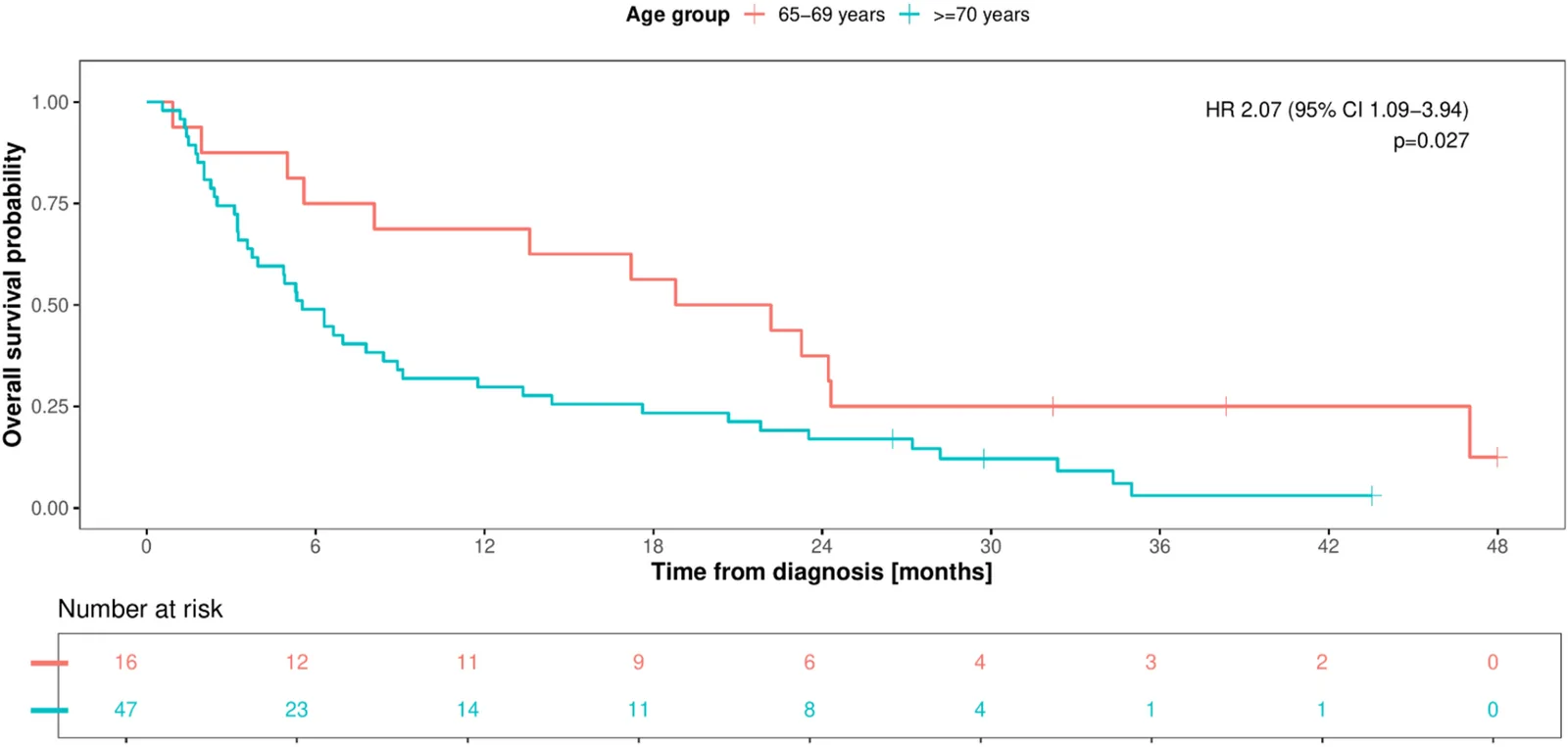
Overall survival stratified by age group. Kaplan-Meier estimates compare patients aged < 70 years with those aged > = 70 years. The displayed hazard ratio (HR) and 95% confidence interval are derived from an univariable Cox proportional hazards model and refer to patients aged > = 70 years relative to those aged < 70 years (HR 2.05, 95% CI 1.10–3.82; *p* = 0.023). Censoring is indicated by crosses, and the number at risk is shown below the plot

**Fig. 4:**
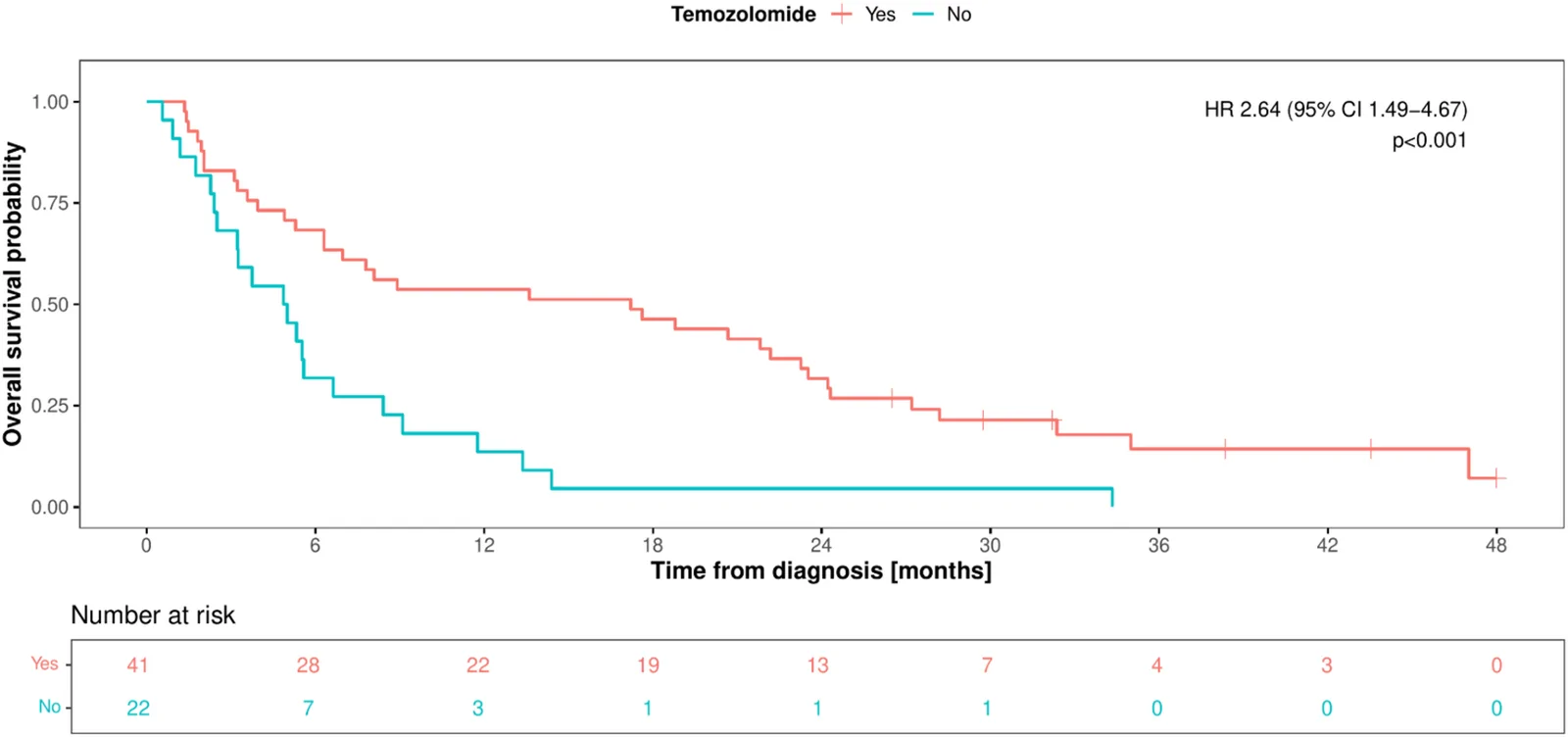
Overall survival stratified by temozolomide administration. Kaplan-Meier estimates compare patients who received temozolomide (TMZ) with those who did not. The displayed hazard ratio (HR) and 95% confidence interval are derived from an univariable Cox proportional hazards model and refer to no TMZ relative to TMZ (HR 2.65, 95% CI 1.53–4.59; *p* < 0.001). This unadjusted association is descriptive and should not be interpreted as a causal treatment effect because of treatment selection and potential confounding by indication. Censoring is indicated by crosses, and the number at risk is shown below the plot

A detailed summary of the univariable and multivariable Cox regression analyses is provided in Table 2. Overall OS is shown in Fig. 1, while Kaplan–Meier curves for OS stratified by age and TMZ administration are presented in Figs. 3 and 4, respectively.

Univariable and multivariable Cox regression results for PFS are summarized in Table 3. The prespecified multivariable model included 56 patients and 53 progression or death events. Increasing age was associated with a higher hazard of progression or death (HR 1.07 per year, 95% CI 1.01–1.14; *p* = 0.027). Female sex was associated with a lower hazard (HR 0.45, 95% CI 0.23–0.89; *p* = 0.022), as was higher KPS (HR 0.98 per point, 95% CI 0.96–1.00; *p* = 0.039). Omission of TMZ was associated with a higher hazard of progression or death (HR 2.34, 95% CI 1.20–4.55; *p* = 0.013). The conventional Cox estimate for GTR was not statistically significant (HR 0.71, 95% CI 0.38–1.32; *p* = 0.275); however, this estimate should be interpreted in light of the non-proportional hazards identified for resection status.

**Table 3:** Univariable and multivariable Cox regression analyses for progression-free survival

Characteristic	Univariable			Multivariable		
	HR	95% CI	*p*-value	HR	95% CI	*p*-value
Age	1.07	1.01–1.12	0.012	1.07	1.01–1.14	0.027
Female sex	0.71	0.41–1.22	0.215	0.45	0.23–0.89	0.022
KPS	0.98	0.96–0.99	0.006	0.98	0.96–1.00	0.039
No TMZ	3.27	1.75–6.12	< 0.001	2.34	1.20–4.55	0.013
GTR	0.72	0.40–1.30	0.269	0.71	0.38–1.32	0.275

HR, hazard ratio; CI, confidence interval; KPS, Karnofsky Performance Status; TMZ, temozolomide; GTR, gross total resection. Univariable and multivariable analyses were performed in the same complete-case cohort (*N* = 56; 53 progression or death events). The multivariable model included age, sex, KPS, TMZ administration, and resection status irrespective of univariable statistical significance. HRs for age and KPS are reported per 1-year and 1-point increase, respectively. Reference categories were male sex, TMZ administration, and non-GTR. For PFS, the proportional hazards assumption was violated for resection status (Schoenfeld test *p* = 0.0065); the conventional Cox estimate for GTR should therefore not be interpreted as a constant effect over time. A time-varying coefficient sensitivity analysis is reported in Supplementary Table S4

Supplementary Kaplan–Meier curves for PFS according to age, TMZ administration, and KPS are shown in Figures S1–S3. Additional OS subgroup analyses are presented in Figures S4A–S4B.

Treatment allocation differed substantially according to baseline characteristics. Patients who did not receive TMZ were older than those who received TMZ (median 77.0 vs. 74.0 years; *p* = 0.028), had substantially lower KPS (median 60 vs. 70; *p* < 0.001), and differed in the radiotherapy regimen received (*p* = 0.005). These differences indicate substantial treatment selection and potential confounding by indication.

### Sensitivity Analyses

No statistically significant violation of the proportional hazards assumption was detected for the OS model (global *p* = 0.259); individual tests were also non-significant, including resection status (*p* = 0.081). For PFS, the global test was not statistically significant (*p* = 0.0951), but resection status showed evidence of non-proportional hazards (*p* = 0.0065), whereas age, sex, KPS, and TMZ did not.

A Cox model incorporating a time-varying coefficient for GTR confirmed that its association with PFS changed over follow-up. GTR was associated with a lower early hazard of progression or death (HR at 1 month 0.11, 95% CI 0.02–0.51; HR at 3 months 0.29, 95% CI 0.11–0.79), with attenuation by 6 months (HR 0.63, 95% CI 0.31–1.30) and thereafter. Estimates at later time points were increasingly imprecise because of the decreasing number of patients at risk.

In the sensitivity analysis restricted to patients with confirmed IDH-wildtype disease, 46 patients were available for complete-case multivariable analysis. The association between TMZ omission and worse outcome remained present for both OS (HR 2.27, 95% CI 1.08–4.76; *p* = 0.031) and PFS (HR 2.30, 95% CI 1.10–4.80; *p* = 0.027), although estimates for several other covariates became less precise because of the reduced sample size.

Additional adjustment for radiotherapy regimen did not materially attenuate the TMZ association. In the subgroup with known MGMT promoter status, an exploratory model simultaneously adjusting for MGMT status and radiotherapy regimen yielded an HR of 3.77 (95% CI 1.46–9.75; *p* = 0.006) for death among patients not receiving TMZ. For PFS, resection status continued to show non-proportional hazards; therefore, the corresponding model incorporated a time-varying coefficient for GTR. In this model, TMZ omission remained associated with a higher hazard of progression or death (HR 3.43, 95% CI 1.42–8.32; *p* = 0.006). These sensitivity analyses support the stability of the observed association but do not eliminate residual confounding or potential bias related to treatment timing.

To further address the definition and timing of TMZ exposure, additional sensitivity analyses classified treatment according to concomitant TMZ administration (Supplementary Table S5). When survival was measured from diagnosis, absence of concomitant TMZ was associated with higher hazards for both OS (HR 2.39, 95% CI 1.21–4.75; *p* = 0.013) and PFS (HR 2.14, 95% CI 1.09–4.22; *p* = 0.027). In the PFS model incorporating a time-varying GTR coefficient, the corresponding HR was 2.03 (95% CI 1.05–3.93; *p* = 0.036). Results were similar when survival was re-anchored at radiotherapy initiation: HR 2.42 (95% CI 1.21–4.82; *p* = 0.012) for OS and HR 2.11 (95% CI 1.07–4.17; *p* = 0.032) for PFS; in the PFS model with a time-varying GTR coefficient, the HR was 1.99 (95% CI 1.02–3.87; *p* = 0.042). These sensitivity analyses reduce concerns related to exposure definition and treatment timing but do not eliminate residual confounding or establish a causal treatment effect.

Detailed proportional hazards test results are provided in Table S3, with corresponding Schoenfeld residual plots shown in Figures S5A–S5B. Time-specific hazard ratios from the time-varying resection model are provided in Table S4. 

## Discussion

In this retrospective single-center real-world cohort of patients aged ≥ 65 years with GBM, median OS was 7.0 months and median PFS was 5.3 months. Increasing age was associated with worse OS and PFS, whereas higher KPS was associated with more favorable PFS. Female sex and TMZ administration were associated with more favorable outcomes in the primary multivariable models. Radiologically confirmed GTR showed a directionally favorable but borderline association with OS, while its association with PFS varied over time. Overall, the findings should be regarded as hypothesis-generating rather than causal.

The observed survival outcomes are broadly consistent with randomized evidence in older patients with GBM. Hypofractionated radiotherapy has shown survival outcomes comparable with conventional radiotherapy while reducing treatment burden [12, 14, 20], and the addition of TMZ to short-course radiotherapy improves survival, particularly in patients with MGMT promoter methylation [13]. In our cohort, hypofractionated radiotherapy was the most frequently used regimen, but treatment selection was not randomized and therefore does not permit conclusions regarding comparative effectiveness. Similar heterogeneity in treatment selection and outcomes has been reported in retrospective and population-based studies of older and very old patients with GBM [15–19].

Increasing age was independently associated with worse OS and PFS. This is consistent with previous studies identifying age as an adverse prognostic factor in GBM [21]. The relatively narrow age range in our cohort may have limited the precision of age-related estimates, particularly for PFS.

The association between female sex and more favorable outcomes warrants cautious interpretation. Previous work has not consistently identified sex as an independent prognostic factor after adjustment for molecular and clinical characteristics [22]. In our cohort, MGMT promoter methylation did not differ significantly by sex among patients with known status (*p* = 0.423). Nevertheless, given the modest sample size and observational design, the observed sex association should be considered exploratory.

TMZ administration was consistently associated with more favorable OS and PFS. This is concordant with randomized evidence for TMZ in GBM and with randomized and real-world evidence in older patients [7, 13, 17]. The association remained present in exploratory analyses restricted to confirmed IDH-wildtype disease and after additional adjustment for MGMT promoter status and radiotherapy regimen. Importantly, sensitivity analyses defining exposure specifically as concomitant TMZ and analyses re-anchoring survival at radiotherapy initiation yielded associations in the same direction. Nevertheless, patients who did not receive TMZ were older, had lower KPS, and differed in radiotherapy regimen, demonstrating substantial confounding by indication. Exact TMZ initiation dates were not consistently available, precluding formal time-dependent exposure modeling. Although the concomitant-TMZ and radiotherapy-start sensitivity analyses reduce concerns related to treatment timing, residual confounding and potential immortal-time bias cannot be fully excluded. The observed association should therefore not be interpreted as a causal treatment effect.

Radiologically confirmed GTR showed a directionally favorable association with OS, although the estimate did not reach conventional statistical significance in the revised primary model (HR 0.52, 95% CI 0.27–1.01; *p* = 0.054). This direction is consistent with previous observational studies in older patients with GBM [15, 17]. For PFS, resection status violated the proportional hazards assumption. The time-varying analysis suggested a pronounced early association of GTR with a lower risk of progression or death that attenuated over follow-up; later estimates were imprecise because of the decreasing number of patients at risk.

Recurrence patterns could be classified in only 17 patients and should therefore be interpreted cautiously. The relatively frequent out-of-field failures among evaluable patients may reflect selection bias and incomplete follow-up imaging rather than the true recurrence pattern of the cohort. Previous studies have predominantly reported local recurrence and emphasized the importance of resection extent and radiotherapy target definition [23–25].

Several limitations should be considered. The retrospective single-center design and modest sample size limit statistical power and external validity. Treatment was not randomly assigned, resulting in substantial potential for confounding by indication, particularly for TMZ administration and radiotherapy regimen. Missing KPS, molecular, volumetric, and recurrence data reduced the effective sample size and may have introduced complete-case selection bias. PFS was based on retrospective clinical and imaging assessment and was therefore more susceptible than OS to measurement error and differences in surveillance intervals. In addition, the study period spanned 2014–2023 and encompassed evolving diagnostic criteria and treatment practices. No standardized geriatric or frailty assessment was available; KPS should therefore not be interpreted as a direct measure of frailty. Strengths include the detailed real-world dataset, inclusion of an older population underrepresented in randomized trials, use of a common diagnosis-based time origin, clinically prespecified multivariable models, and transparent sensitivity analyses addressing missing molecular information and non-proportional hazards. Because patients were identified through the Department of Radiation Oncology, the cohort may underrepresent patients who were never referred for radiotherapy or who received supportive care alone. The study period also spanned evolving WHO diagnostic classifications; no centralized neuropathological re-review or retrospective molecular reclassification was possible, and IDH status remained unknown in a subset of patients. Progression was based on contemporaneous routine clinical and imaging assessment rather than a centralized RANO-based review, and treatment-related imaging changes could not be systematically reclassified retrospectively. Finally, exact TMZ initiation dates were not consistently available, and potential immortal-time bias in analyses using binary TMZ exposure cannot be excluded.

Future multicenter studies should prospectively incorporate standardized geriatric assessment, molecular characteristics, treatment-selection variables, and longitudinal imaging to permit more precise evaluation of interactions between age, performance status, MGMT status, resection, and treatment strategy.

## Conclusions

In this retrospective real-world cohort of patients aged ≥ 65 years with GBM, OS and PFS remained limited. Increasing age was associated with worse outcomes, whereas higher KPS was associated with more favorable PFS. Female sex showed favorable associations in the multivariable models, while the association between GTR and PFS varied over time. TMZ administration was consistently associated with more favorable OS and PFS, including in sensitivity analyses based on concomitant TMZ exposure and survival measured from radiotherapy initiation. However, substantial treatment selection and potential residual confounding preclude causal interpretation. These findings should therefore be considered hypothesis-generating and warrant validation in larger prospective cohorts incorporating standardized geriatric assessment.

## Supplementary Information

Below is the link to the electronic supplementary material.

Supplementary Material 1: Tables S1–S5, Figures S1–S6.

## Data Availability

The data that support the findings of this study are derived from institutional cancer registry records and are not publicly available due to privacy and data protection regulations. Access to the data is restricted under German data protection law and the conditions of the ethics approval. Aggregated or anonymized data may be made available from the corresponding author upon reasonable request and with appropriate institutional permissions.

## Abbreviations

CI: Confidence Interval
CTV: Clinical Target Volume
ECOG: Eastern Cooperative Oncology Group Performance Status
EGFR: Epidermal Growth Factor Receptor
GBM: Glioblastoma
GTR: Gross Total Resection
GTV: Gross Tumor Volume
Gy: Gray
HR: Hazard Ratio
IAEA: International Atomic Energy Agency
IDH: Isocitrate Dehydrogenase
IQR: Interquartile Range
KPS: Karnofsky Performance Status
MGMT: O^6-Methylguanine-DNA Methyltransferase
MRI: Magnetic Resonance Imaging
NTR: Near-Total Resection
OS: Overall Survival
PFS: Progression-Free Survival
RT: Radiotherapy
SD: Standard Deviation
STR: Subtotal Resection
TMZ: Temozolomide
TTFields: Tumor Treating Fields
VEGF: Vascular Endothelial Growth Factor
WHO: World Health Organization
