# Supplementary material for: Real-world evidence on the management and outcomes of glioblastoma in elderly patients: a monocentric retrospective analysis
Source: BMC Geriatr. 2026 Oct 13;26:1296. doi: 10.1186/s12877-026-08457-9 (PMC13663175; doi:10.1186/s12877-026-08457-9)
Supplement: Supplementary file 1 [file 12877_2026_8457_MOESM1_ESM.docx]

**Supplementary Material**

Supplementary Table S1. Clinical characteristics according to temozolomide administration

| **Characteristic** | **TMZ yes (N=41)** | **TMZ no (N=22)** | **p-value** |
| --- | --- | --- | --- |
| Age, years, median (IQR) | 74.0 (68.0-75.0) | 77.0 (73.0-79.0) | 0.028 |
| Sex, n (%) |  |  | 0.192 |
| Male | 26 (63%) | 10 (45%) |  |
| Female | 15 (37%) | 12 (55%) |  |
| Karnofsky Performance Status, median (IQR) | 70 (60-80) | 60 (40-60) | <0.001 |
| Missing, n | 4 | 1 |  |
| MGMT promoter status, n (%) |  |  | 0.186 |
| Unmethylated | 11 (27%) | 11 (50%) |  |
| Methylated | 25 (61%) | 9 (41%) |  |
| Unknown | 5 (12%) | 2 (9%) |  |
| Radiologically confirmed resection status, n (%) |  |  | 0.545 |
| Non-GTR | 30 (75%) | 13 (65%) |  |
| GTR | 10 (25%) | 7 (35%) |  |
| Missing, n | 1 | 2 |  |
| Radiotherapy regimen, n (%) |  |  | 0.005 |
| Hypofractionated | 25 (61%) | 16 (73%) |  |
| Normofractionated | 14 (34%) | 1 (5%) |  |
| Ultrahypofractionated | 2 (5%) | 5 (23%) |  |

*Values are median (interquartile range) or n (%). P values are from Wilcoxon rank-sum tests for continuous variables and Fisher exact tests for categorical variables. The differences in age, performance status, and radiotherapy regimen support substantial confounding by indication in analyses of temozolomide.*

Supplementary Table S2. Selected clinical characteristics according to sex

| **Characteristic** | **Male (N=36)** | **Female (N=27)** | **p-value** |
| --- | --- | --- | --- |
| Age, years, median (IQR) | 73.5 (68.0-76.0) | 74.0 (71.5-79.0) | 0.102 |
| Karnofsky Performance Status, median (IQR) | 70 (60-70) | 60 (40-70) | 0.043 |
| Missing, n | 3 | 2 |  |
| MGMT promoter status among known cases, n (%) |  |  | 0.423 |
| Unmethylated | 13/29 (45%) | 9/27 (33%) |  |
| Methylated | 16/29 (55%) | 18/27 (67%) |  |
| MGMT status unknown, n | 7 | 0 |  |
| Temozolomide, n (%) |  |  | 0.192 |
| Yes | 26 (72%) | 15 (56%) |  |
| No | 10 (28%) | 12 (44%) |  |
| Radiologically confirmed resection status, n (%) |  |  | 0.145 |
| Non-GTR | 28 (80%) | 15 (60%) |  |
| GTR | 7 (20%) | 10 (40%) |  |
| Missing, n | 1 | 2 |  |
| Radiotherapy regimen, n (%) |  |  | 0.748 |
| Hypofractionated | 22 (61%) | 19 (70%) |  |
| Normofractionated | 10 (28%) | 5 (19%) |  |
| Ultrahypofractionated | 4 (11%) | 3 (11%) |  |

*For the MGMT comparison, the Fisher exact test was restricted to patients with known MGMT promoter status (p=0.423). All seven patients with unknown MGMT status were male. Other P values correspond to the displayed group comparisons.*

Supplementary Table S3. Proportional hazards assumption assessed using Schoenfeld residuals

| **Covariate** | **OS chi-square** | **OS p-value** | **PFS chi-square** | **PFS p-value** |
| --- | --- | --- | --- | --- |
| Age | 0.109 | 0.742 | 0.614 | 0.433 |
| Sex | 0.187 | 0.665 | 0.064 | 0.800 |
| Karnofsky Performance Status | 1.553 | 0.213 | 2.544 | 0.111 |
| Temozolomide | 0.041 | 0.840 | 0.003 | 0.957 |
| Resection status | 3.049 | 0.081 | 7.417 | 0.0065 |
| GLOBAL | 6.524 | 0.259 | 9.373 | 0.0951 |

*No statistically significant violation was detected in the overall survival model. In the progression-free survival model, resection status violated the proportional hazards assumption (p=0.0065), while the global test was not statistically significant (p=0.0951). This motivated an additional Cox model with a time-varying coefficient for gross total resection.*

Supplementary Table S4. Time-varying association between gross total resection and progression-free survival

| **Time from diagnosis** | **HR for GTR vs non-GTR** | **95% CI** |
| --- | --- | --- |
| 1 month | 0.11 | 0.02-0.51 |
| 3 months | 0.29 | 0.11-0.79 |
| 6 months | 0.63 | 0.31-1.30 |
| 9 months | 1.03 | 0.51-2.10 |
| 12 months | 1.48 | 0.67-3.28 |
| 18 months | 2.49 | 0.91-6.86 |
| 24 months | 3.64 | 1.09-12.19 |

*Hazard ratios are derived from the Cox model including a time-varying coefficient for gross total resection. The early association attenuated over follow-up. Estimates at later time points are based on small numbers at risk and should not be interpreted as evidence of a harmful effect of resection.*

Supplementary Table S5. Sensitivity analyses using concomitant temozolomide exposure and radiotherapy-start time origin

| **Time origin** | **Outcome** | **Model** | **N** | **Events** | **HR: no concomitant TMZ vs concomitant TMZ** | **95% CI** | **p-value** |
| --- | --- | --- | --- | --- | --- | --- | --- |
| Diagnosis | OS | Conventional Cox model | 56 | 50 | 2.39 | 1.21-4.75 | 0.013 |
| Diagnosis | PFS | Conventional Cox model | 56 | 53 | 2.14 | 1.09-4.22 | 0.027 |
| Diagnosis | PFS | Cox model with time-varying GTR coefficient | 56 | 53 | 2.03 | 1.05-3.93 | 0.036 |
| Radiotherapy start | OS | Conventional Cox model | 56 | 50 | 2.42 | 1.21-4.82 | 0.012 |
| Radiotherapy start | PFS | Conventional Cox model | 56 | 53 | 2.11 | 1.07-4.17 | 0.032 |
| Radiotherapy start | PFS | Cox model with time-varying GTR coefficient | 56 | 53 | 1.99 | 1.02-3.87 | 0.042 |

*Hazard ratios compare no concomitant temozolomide (TMZ) with concomitant TMZ and are adjusted for age, sex, Karnofsky Performance Status, and gross total resection (GTR). PFS models with a time-varying GTR coefficient were fitted because resection status violated the proportional hazards assumption. Re-anchoring survival at radiotherapy initiation served as an additional sensitivity analysis addressing treatment-timing bias.*

Figure S1. Progression-free survival according to age group. Kaplan-Meier estimates are shown for patients aged 65-69 years and >=70 years, with numbers at risk displayed below the survival curves.

Figure S2. Progression-free survival according to temozolomide administration. Kaplan-Meier estimates are shown for patients who did and did not receive temozolomide, with numbers at risk displayed below the survival curves. Because treatment groups differed substantially in age, performance status, and radiotherapy regimen, the observed association should not be interpreted as a causal treatment effect.

Figure S3. Progression-free survival according to Karnofsky Performance Status (KPS). Kaplan-Meier estimates are shown for KPS <70% and >=70%, with numbers at risk displayed below the survival curves.

Figure S4A. Overall survival according to sex. Kaplan-Meier estimates are shown for male and female patients, with numbers at risk displayed below the survival curves.

Figure S4B. Overall survival according to radiologically confirmed resection status. Kaplan-Meier estimates are shown for gross total resection (GTR) and non-GTR, with numbers at risk displayed below the survival curves.

Figure S5A. Assessment of the proportional hazards assumption for overall survival. Scaled Schoenfeld residual plots are shown for the covariates included in the final multivariable Cox model. The global Schoenfeld test was not statistically significant (p=0.259).

Figure S5B. Assessment of the proportional hazards assumption for progression-free survival. Scaled Schoenfeld residual plots are shown for the covariates included in the final multivariable Cox model. Resection status showed evidence of non-proportional hazards (p=0.0065), while the global Schoenfeld test was not statistically significant (p=0.0951). This finding motivated the time-varying coefficient analysis reported in Supplementary Table S4.

**Figure S6. Patient selection and final primary study cohort.** Of 71 patient records identified in the radiation oncology database, three patients with documented IDH-mutant tumors and one patient treated with stereotactic radiotherapy were initially excluded. Four additional records were excluded because of irreconcilable chronological inconsistencies in the available retrospective treatment or follow-up data that could not be verified from the available source documentation, resulting in a final primary study cohort of 63 patients.
